# Case report: Dendritic cell-cytokine induced killer cell therapy in subjects with chronic lymphocytic leukemia and peritoneal cancer

**DOI:** 10.3389/fmed.2023.1240330

**Published:** 2023-10-09

**Authors:** Brian Mehling, DongCheng Wu, Ellen O’Gorman, Daniel Sheridan, Doreen Santora, Renata Mihályová

**Affiliations:** ^1^BHI Therapeutic Sciences Inc., Hackensack, NJ, United States; ^2^Department of Biochemistry and Molecular Biology, Wuhan University School of Basic Medical Sciences, Wuhan, China; ^3^Blue Horizon International, Bratislava, Slovakia

**Keywords:** immunotherapy, cancer, dendritic cells, cytokine-induced killer cells, leukemia, peritoneal cancer

## Abstract

This study aimed to characterize the safety and efficacy of DC-CIK therapy in two patients with previously treated chronic lymphocytic leukemia or peritoneal cancer, respectively. Participants had received conventional chemotherapy treatment for their specific cancers, and in addition, 1–2 treatments of DC-CIK therapy were administered to subjects over the course of 1 year. Subject A received an initial dosage of 3 intravenous infusions of DC-CIK therapy on three successive days and a repeat dosage 6 months later. Subject B received an initial dosage of 3 intravenous infusions of DC-CIK therapy on three successive days and received further chemotherapy after approximately 1 year. No treatment-related adverse events were reported, and both patients experienced favorable outcomes from the treatment, including enhanced treatment response, increased chemotherapy tolerance, and prolonged survival in comparison to typical 5-year survival rates.

## 1. Introduction

Dendritic cells (DCs) are antigen-presenting cells of the immune system. Their functions are to capture and process tumor antigens, express lymphocyte costimulatory molecules, and secrete cytokines to initiate immune responses (1, 2). Cytokine-induced killer (CIK) cells represent a unique population of cytotoxic T lymphocytes (CTL) with the characteristic CD3 + CD56 + phenotype (3). CIK cells activated by dendritic DC stimulation show increased anti-tumor activity, and several studies indicate that DC/CIK therapy has potential benefits for subjects with various forms of cancer with no apparent side effects (2).

Clinical trials have suggested that DC-CIK therapy can work as an adjunct alongside chemotherapy to support the production of tumor-reducing cytokines and subsequently slow tumor progression (4–6). Meta-analysis of the clinical application of DC-CIK in various malignancies has demonstrated promising results across a range of cancers, and a systematic review of 17 randomized clinical studies, including 1,172 patients with advanced cancer, highlighted how DC-CIK treatment improved the median survival time, progression-free survival and time to progression when delivered in conjunction with chemotherapy (2, 7). Specific to leukemia, Zheng et al. investigated the clinical efficacy and safety of DC-CIK therapy combined with chemotherapy in eliminating minimal residual leukemia (8). Patients with acute leukemia received either chemotherapy only or combined DC-CIK therapy and chemotherapy. The combined group experienced a 45.8% rate of molecular biological remission in comparison to a mere 8% in the chemotherapy-only group. In another trial, patients with acute myeloid leukemia who received DC-CIK infusions every 3 months for 2–4 cycles demonstrated a 5-year overall survival rate of 90.5% with a relapse-free survival rate of 65.2% (9).

With the preclinical trial evidence, alongside the clinical trial data available, we hypothesized that treatment with DC-CIK immunotherapy as an adjunct to chemotherapy would improve patient outcomes in two forms of cancer: peritoneal cancer, and chronic lymphocytic leukemia. This is the first recorded DC-CIK treatment of either cancer available in the current literature.

## 2. Case presentations

Two female subjects were treated at Nemocnica Malacky Hospital (Malacky, Slovakia) for differing cancer diagnoses between April 2016-May 2020. Informed consent was obtained through the signature of an informed consent form from both patients during the enrollment stage of the study, with written consent obtained from each subject to publish the potentially identifiable data contained in the report. The study was approved by the Institutional Review Board of the Institute of Regenerative and Cellular Medicine (IRCM-2021-308). All procedures followed were in accordance with the ethical standards of the responsible committee on human experimentation (institutional and national) and with the Helsinki Declaration of 1975, as revised in 2000.

Both subjects received DC-CIK as an adjunct to traditional therapy. The subject’s mononuclear cells, including DC and T-cells, were expanded *in vitro* following treatment with GM-CSF, IL-4, and TNFα cytokines. The expanded DC-CIK cell population was subsequently cultured with the patient-specific tumor antigen to immunologically prime the DC-CIK therapy to target the patient’s cancer.

### 2.1. Subject A

**FIGURE 1:**
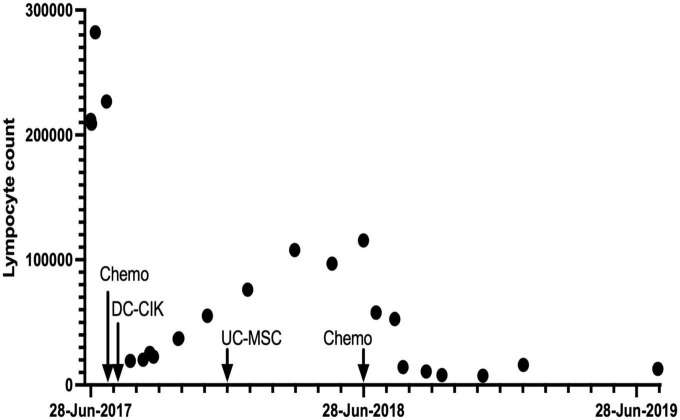
Leukocyte counts/mL for Patient A over a 24-month period.

Subject A was an 89 year old female diagnosed with classic chronic lymphocytic leukemia (CLL) of the B-cell series in July 2017 (Table 1). The patient presented at Binet Stage C, with anemia, thrombopenia, and bone marrow infiltration over 80%. Blood marker testing demonstrated deranged leukocyte numbers measuring over 200,000 cells per microliter. Two weeks later, the patient underwent chemotherapy with 56 mg of Bendamustine/Rituximab. Due to the tumor load and to preserve cytokine reaction, the first cycle was completed without Rituximab. Following this, the patient provided the necessary blood donation to develop the immunotherapy treatment. The patient then received three intravenous infusions of DC-CIK therapy on three successive days. Each dose was 50 mL and contained 5.59 × 10^7^ cells per 5 mL, 8.65 × 10^7^ cells per 5 mL, and 1.4 × 10^8^ cells per 5 mL, respectively. Weekly laboratory blood tests were completed for 4 weeks to monitor for change. Leukocyte numbers dropped significantly post-treatment and remained in the 20,000–30,000-figure range until a gradual increase began in December 2017 (Figure 1). There were no adverse effects reported directly related to the immunotherapy administration. The subject received one intravenous infusion of umbilical cord-derived mesenchymal stem cells at a dose of 1.3 × 10^9^ cells per 5 mL. Collection, isolation of MSCs from human umbilical cord blood, and quality control testing were performed according to methods described by Mehling et al. (10). Following this, leukocyte numbers continued to rise until June 2018, when the patient underwent a further 6 sessions of chemotherapy. Immediately post-chemotherapy, the patient’s leukocyte numbers declined by half and proceeded to drop within the normal range for several months before settling just outside the upper end of the normal range. As of 2022, the patient is still alive without recurrence of the disease.

**TABLE 1:** Table detailing clinical course of Subject A.

Date	Reported findings
Jun-17	Blood draw in preparation for stem cell treatment demonstrates alarming leukocytes at 221,000 Transfer to hospital with suspicion of chronic lymphocytic leukemia. Repeat blood test reveals leukocytes at 209,000
19 July-17	CT scan and bone marrow biopsy Diagnosis of classic chronic lymphocytic leukemia of the B-cell series Binet stage C
26 July-17	Chemotherapy with 70% reduced dose 56 mg Bendamustine/Rituximab First cycle without rituximab to prevent cytokine reaction
Aug-17	DC-CIK therapy Current leukocytes at 96,350
June-18	6 sessions of chemotherapy: Bendamustine 60 mg, Granisetron, Dexamethasone, Pantoprazole, and Ondansetron

### 2.2. Subject B

Subject B was 70 years of age when diagnosed with secondary-malignant neoplasm of retroperitoneum and peritoneum in November 2015 (Table 2). CT and colonography confirmed peritoneal carcinoma and ovaries with extensive pathological changes of the peritoneum, indicating metastatic disease. Furthermore, there was free fluid in the abdomen and pathological infiltrates pressing on the lower part of the colon transversum. The patient underwent four cycles of chemotherapy from January to March 2016. In April, the patient provided the required blood donation to prepare the DC-CIK therapy infusions. The patient then received three intravenous infusions of DC-CIK therapy on three successive days. Cell dosage consisted of 2.3 × 10^9^, 2.54 × 10^9^, and 2.58 × 10^9^ cells per 5 mL, respectively, delivered in a 50 cc saline infusion. There were no adverse effects reported related to the immunotherapy administration.

**TABLE 2:** Table detailing clinical course of Subject B including reported CT.

Date	Reported findings
Nov-15	Peritoneal carcinoma and ovaries Free fluid in the abdomen Extensive pathological changes of the peritoneum Pathological infiltrates pressing on the lower part of the colon
Jan-Mar-16	Four cycles of chemotherapy
Apr-16	DC-CIK therapy
May-16	Significant regression of previous findings Size regression of the hypodense formation in the left ovary Pathological infiltrates urging the peritoneum Degenerative changes in the lumbar spine
Jun-16	6 cycles of chemotherapy and 4 cycles of bevacizumab Radical hysterectomy and omentectomy
Jan-17	DC-CIK therapy
Mar-17	New lesion under left abdominal wall
May-18	Significant progression of the underlying disease Increasing several peritoneal metastases New metastases in left abdominal wall Hypermetabolic lesion in the liver Malignant lymph nodes in the left pelvic region Further peritoneal metastases
Nov-18	New peritoneal metastases in the hypogastrium Implant metastases at pyloric level Metastases sub diaphragmatically at S7 liver segment level Metastases in the spleen
Nov-18	Chemotherapy
Jun-19	Interstitial changes in the upper lung lobe Carcinomatous Lymphangiopathy Interstitial inflammation and fibrosis Size progression of previous peritoneal metastases New liver metastases Further growth of splenic lesion Size progression of metastases in left abdominal cavity wall
May-20	Significant morpho metabolic progression of disseminated metastatic involvement of visceral peritoneum, mesentery, anterior left abdominal wall and spleen

In May, the patient underwent blood testing, CT, and oncological review by the treating medical team. Blood counts remained unchanged, and CT findings included significant regression of findings, including size regression of the hypodense formation in the left ovary. The patient then underwent six cycles of chemotherapy and four cycles of bevacizumab. It was reported that Subject B tolerated chemotherapy better with fewer side effects and, as a result, was able to undergo further surgery. In August, a radical hysterectomy and omentectomy were completed alongside further chemotherapy. The patient received a follow-up dosing regimen of DC-CIK therapy in January 2017. Cell dosage consisted of 4.08 × 10^8^, 2.71 × 10^8^, and 3.35 × 10^8^ cells per 5 mL, respectively. Over the following months, repeated CT imaging demonstrated a gradual progression of the underlying disease with increasing peritoneal metastases, malignant lymph nodes in the left pelvis, and further metastases in the spleen. It is important to note that at this stage in treatment, it was reported that the treating oncologist discouraged the patient from engaging in the trial further due to misconceptions and a lack of confidence in the immunotherapy treatment. In June 2019, there were interstitial changes in the upper lung lobes with a differential diagnosis of carcinomatous lymphangiopathy with interstitial inflammation and fibrosis. Following a diagnosis of a new hypermetabolic focus in the tail of the pancreas, the patient declined further active treatment, and a palliative care pathway was initiated. However, as of late 2022, almost 5 years after the initial diagnosis, Subject B remains alive.

## 3. Diagnostic assessment

Prior to treatment, subjects underwent screening and baseline assessments (inclusion/exclusion criteria, physical examination) and examination of medical records. Prior to scheduling, the subject’s medical history and records were examined by the investigator. Once enrolled, the EORTC QLQ-C30 questionnaire, disease-specific questionnaires, serological tumor markers test, the percentage of Treg cells in the peripheral blood, ultrasound, or X-ray, were administered.

Subjects were tracked for 3 years to assess the safety of DC-CIK therapy and to evaluate its effect on cancer therapy. At the end of 3rd-year final questionnaires and tests (EORTC QLQ-C30 and disease-specific questionnaires, serological tumor markers, and the percentage of Treg cells in the peripheral blood, X-ray, or ultrasound) will be administered to assess the overall safety of DC-CIK therapy and effect on cancer therapy.

## 4. Discussion

The case report describes two patients with different forms of cancer who achieved positive results when treated with a combination of chemotherapy and DC-CIK immunotherapy. As far as we are aware, this is the first report of DC-CIK therapy for CLL and peritoneal cancer, respectively. The use of DC-CIK as an adjunct to standard cancer treatments has piqued the interest of oncologists due to convincing results reported by preclinical and clinical trials in several different cancer types (9, 11–14). Research investigating a combination of DC-CIK immunotherapy and chemotherapy in different cancer types has revealed significant variations in cell dosage. A consistent, standardized dosage has not been identified. In this study, the number of cells per 5 mL varied even across each intravenous administration, with the patients receiving a range from 5.59 × 10^7^ to 2.58 × 10^9^ per dose. Other studies have utilized a range of dosages and treatment regimens such as four treatments of 1.3 × 10^9^ cells intervals of a month (15, 16), 1.2 × 10^10^ for 18 cycles (15) or 1.27 × 10^7^ every second day for 6 days (17). With varying degrees of success across different trials alongside the promising results achieved in this trial with varying doses, further investigation into a standardized dosing regime is warranted.

Subject B notably experienced a reduction in chemotherapy side effects and increased tolerability of the chemotherapy, which further allowed her to undergo significant surgery with curative intent. This has been a trend reported in DC-CIK immunotherapy trials whereby the adverse effects of chemotherapy can be significantly reduced by this treatment. It is thought that the treatment reduced leukopenia, peripheral neuritis, gastrointestinal side effects, liver dysfunction, and myelosuppression (2, 7, 13). With a myriad of side effects and often high levels of toxicity, there is a high degree of balancing between efficacy and toxicity that must be considered by oncologists (18–20). Apart from severe adverse effects and generalized weakening of the patient, the patient’s immune response can even go as far as limiting the tumor-targeting effects of the treatment through systemic or localized responses (18). Undeniably, a reduction in adverse effects associated with chemotherapy greatly improves the quality of life of cancer patients and thus provides a strong case for the incorporation of DC-CIK therapy into the treatment regimen of cancer patients.

There is a significant pathophysiological difference between the cancer of the two subjects, specifically the contrast between solid and liquid cancers. Both the solid and liquid cancer subjects in this trial experienced positive outcomes in their overall cancer treatment, echoing results demonstrated by previous research in both groups (9, 11, 13, 14). Notably, in this trial and in numerous other aforementioned systematic reviews and meta-analyses of DC-CIK clinical trials, there have been no severe adverse effects reported related to the DC-CIK immunotherapy. This supports the safety and tolerability of utilizing this therapy as an adjunct to all cancer treatments.

However, the significant disparities between the two patients in this study also present a limitation to the work. It is difficult to ascertain specific trends or model the generalizability of this treatment on either solid or liquid cancers due to the heterogeneity of the cancer types. In addition, it is challenging to isolate the contribution of the DC-CIK therapy to the therapy when dealing with diverse cancer types. Other factors related to the cancer type should be considered, such as its aggressiveness, stage, and response to standard treatments.

Only around 65% of patients over 80 years are expected to survive for 5 years or more after a diagnosis of CLL (20, 21). This number drops further with increasing Binet Stage of disease, with survival figures decreasing further with Stage B or C disease. Some literature estimates the survival time as 2–3 years for patients with Stage C CLL (22, 23). Crucially, in this study, Subject A was considered very high risk due to her age and disease severity at Stage C. Despite this, at the time of writing, Subject A was disease-free for 4 years post-treatment. It is difficult to ascertain the specific impact of the DC-CIK on the patient’s recovery. However, considering the expected prognosis that the patient has exceeded, alongside the risk factors and severe disease, it is reasonable to assume that the DC-CIK therapy had some enhancing effects on the treatment.

As we are aware, this is the first reported case of the treatment of peritoneal cancer with DC-CIK. Although Subject B’s primary tumor was considered peritoneal, she also suffered from ovarian, pancreatic, and liver metastases, all of which have demonstrated promising results when treated with DC-CIK in previous research (17, 24, 25). Notably, a recent clinical trial comparing a combination of DC-CIK with chemotherapy or chemotherapy alone for the treatment of ovarian cancer demonstrated greatly enhanced clinical efficacy, enhanced immune function, and reduced levels of serum tumor markers in the DC-CIK group (25). Although there is a paucity of research specific to peritoneal cancer, the clinical trajectory of Subject B fits with the benefits and success achieved in previous trials for other solid cancer types.

In this report, we describe the case of two female patients with previously treated lymphocytic leukemia or peritoneal cancer, respectively, who received DC-CIK immunotherapy. The patients presented with different manifestations of cancer yet experienced similar outcomes on their treatment journey. Neither patient experienced adverse effects directly related to the DC-CIK therapy, and one patient reported significantly fewer side effects from chemotherapy post-immunotherapy administration. Subject A exceeded the 5-year survival rate for patients with her degree of disease despite being markedly older in age. Subject B is the first reported case of DC-CIK treatment for primary peritoneal cancer, and despite initiating a palliative care pathway in 2017, she remains alive 5 years later. In line with previous research, this case report supports the usage of DC-CIK immunotherapy in conjunction with standard cancer treatment and chemotherapy and highlights the need for further research and development of accessible DC-CIK therapy.

## Data availability statement

The original contributions presented in this study are included in this article/supplementary material, further inquiries can be directed to the corresponding author.

## Ethics statement

Written informed consent was obtained from each subject to publish the potentially identifiable data contained in the report.

## Author contributions

BM conceived, designed the study, and treatment protocol. RM set up the study, obtained the ethical approval, managed the patients, and collected data. EO’G and DoS analyzed the data, interpreted the results, and wrote the manuscript. All authors read and approved the final version of the manuscript.
